# Safety of a tetravalent live dengue virus vaccine in children responding to one serotype only

**DOI:** 10.1172/jci.insight.200741

**Published:** 2026-03-17

**Authors:** Laura J. White, Lindsay D. Hein, Maria Abad Fernandez, Cameron Adams, Elizabeth Adams, Emily Freeman, Ruby Shah, Lakshmanane Premkumar, Kristal An Agrupis, Maria Vinna Crisostomo, Jedas Veronica Daag, Michelle Ylade, Jacqueline Deen, Ana Lena Lopez, Leah Katzelnick, Aravinda M. de Silva

**Affiliations:** 1 Department of Microbiology and Immunology, University of North Carolina at Chapel Hill, North Carolina, USA.; 2 Institute of Child Health and Human Development, University of the Philippines Manila, Manila, Philippines.; 3 Viral Epidemiology and Immunity Unit, Laboratory of Infectious Diseases, National Institute of Allergy and Infectious Diseases, National Institutes of Health, Bethesda, Maryland, USA.

**Keywords:** Immunology, Infectious disease, Virology, Vaccines

## Abstract

Dengue virus (DENV) vaccines should be designed to induce balanced protective immunity to all 4 DENV serotypes to mitigate the risk of vaccine-enhanced dengue disease. The first tetravalent live DENV vaccine (Dengvaxia) tested in humans was efficacious in children who were partially immune to DENV at baseline. In DENV-naive children, the vaccine was not efficacious and placed some children at risk of more severe WT DENV breakthrough infections. To define dengue vaccine responses at the individual patient level and their relationship to mild and severe dengue infections, we prospectively studied a cohort of DENV-naive children who received 1 dose of Dengvaxia. The vaccine stimulated variable responses that neutralized 0, 1 (monotypic), or 2+ (multitypic) serotypes in individual children. Using a logistic regression model, we found that vaccinated children with neutralizing antibody (NAb) to 1 serotype only were at greater risk developing dengue compared with children who were not vaccinated (odds ratio 5.07). This risk was not observed in vaccinated children with no NAb or NAb to 2 or more serotypes. We propose that individuals with durable NAb to 1 serotype have an abundance of serotype cross-reactive, nonneutralizing antibodies implicated in the enhanced replication of heterologous serotypes.

## Abstract 2

Some children receiving an experimetnal vaccine experienced more severe dengue infections. This study has revealed properties of vaccine responses linked to severe infections.

## Graphical abstract

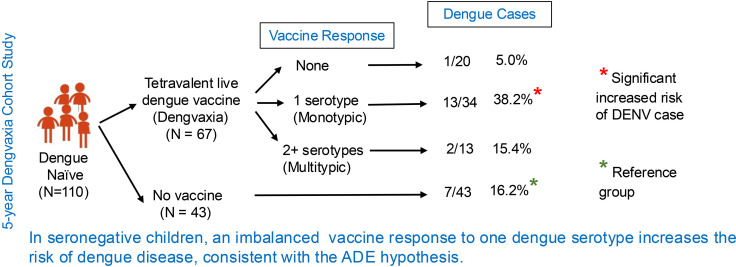

## Introduction

The 4 dengue virus (DENV1-4) serotypes are mosquito-borne flaviviruses that infect several hundred million people each year in tropical and subtropical regions of the world (1, 2). Vaccination is an effective method for preventing flavivirus infections, as demonstrated by existing highly effective vaccines against yellow fever virus (YFV) and Japanese encephalitis virus (JEV). Unlike YFV and JEV vaccines, DENV vaccines must provide tetravalent protection against the 4 serotypes because immunity to a single serotype has been linked to antibody-enhanced replication of heterologous serotypes, leading to more severe clinical disease (3, 4). Here we report on studies conducted with a live-attenuated DENV vaccine (Dengvaxia) to reveal properties of vaccine-stimulated antibodies linked to more severe breakthrough infections.

Leading DENV vaccines are based on tetravalent live-attenuated virus formulations (5). The 2 leading candidates (Dengvaxia and Qdenga) suffer from uneven replication of vaccine viruses, resulting in imbalanced immunity to 1 or 2 serotypes only (6–9). In Dengvaxia clinical trials, in children with no prior immunity to DENV, the vaccine was effective against DENV4 but not serotypes 1, 2, and 3 (10–12). Among seronegative children enrolled in Qdenga clinical trials, investigators have reported high efficacy against DENV2 and partial or no efficacy against the other 3 serotypes (13–15). These observations, together with other preclinical and laboratory studies, indicate that—while Dengvaxia and Qdenga were formulated as tetravalent vaccines—in practice, only the DENV4 or DENV2 vaccine components reliably replicated in each vaccine, respectively (9, 16–18). In children with preexisting DENV immunity, both vaccines stimulated secondary immune responses with broad efficacy across serotypes. In DENV-naive children, vaccine responses and efficacy were strongly biased in favor of the single, dominant replicating vaccine serotype in each vaccine.

In 2016, the World Health Organization recommended the use of Dengvaxia in children 9 years and older living in countries with a high burden of dengue (19). The Philippines and Brazil initiated mass vaccination of older children in selected regions. A secondary analysis of Dengvaxia clinical trial data revealed that seronegative children who were vaccinated and experienced breakthrough DENV infections were at greater risk of being hospitalized with severe dengue compared with unvaccinated children experiencing DENV infections (12, 20). While researchers had been aware of the possibility of vaccine failure against a specific serotype leading to more severe WT DENV infections, these findings raised the level of this threat and changed the research and regulatory landscape for dengue vaccines (21, 22). The mass vaccination program in the Philippines was suspended when the company reported that the vaccine was contraindicated in those seronegative for DENV (DENV seronegative individuals). While the secondary analysis of Dengvaxia clinical trial data demonstrated an increased risk of severe dengue disease in seronegative vaccinated children, the studies did not provide any granularity at the individual patient level on the properties of vaccine-stimulated immunity linked to protective, neutral, or more severe outcomes.

**Figure 1:**
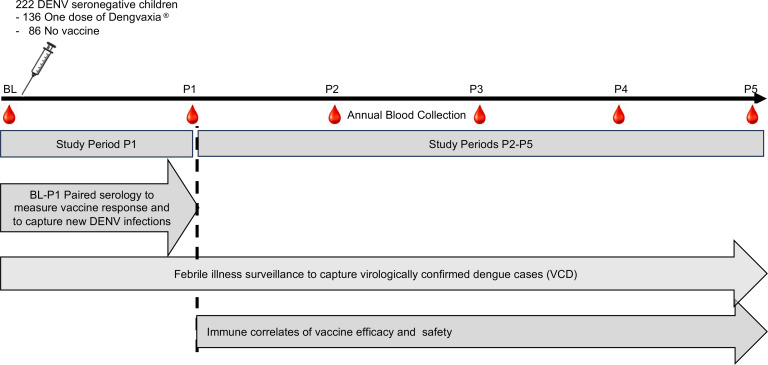
A 5-year prospective cohort study to evaluate the performance of a live attenuated dengue virus vaccine in baseline seronegative children. During mass immunization of children (ages 9–14 years) with Dengvaxia in the Philippines in 2017, 2,996 children (60% vaccinated) were recruited in Cebu province to determine the immunogenicity, safety, and efficacy of the vaccine by baseline dengue serostatus. The children were prospectively monitored for fever to capture symptomatic dengue infections. Blood samples were collected from the entire cohort at the indicated follow-up times (P1–P5) to measure vaccine immunogenicity and to capture all dengue infections (asymptomatic and symptomatic) by serology. P1 blood samples were collected approximately 20 months after vaccination (median = 20 months, IQR 17-22 months). The focus of the current study consist of (a) paired blood samples collected at BL and P1 from 222 children (136 vaccinated and 86 not vaccinated) who were dengue-naive (seronegative) at enrollment to characterize each individual’s response to the vaccine and to capture all new DENV infections (asymptomatic and symptomatic) in the vaccine and no vaccine groups, and (b) symptomatic DENV infections (VCDs) captured by fever surveillance from P1 to P5. In particular, we focus on individual level vaccine responses at P1 and vaccine breakthrough VCDs that occurred between P1 and P5 to identify vaccine responses correlated with protection and vaccine enhanced dengue disease.

When Dengvaxia was first introduced as a pediatric vaccine in the Philippines in 2016, we established a cohort of 2,996 children (ages 9–14) in Cebu, Philippines and prospectively followed the cohort for 5 years to study the immunogenicity and efficacy of Dengvaxia by baseline (BL) dengue serostatus (Figure 1) (23). Sixty percent of the children received 1 dose of the vaccine, and the remaining 40% were not vaccinated. While Dengvaxia was approved as a 3-dose vaccine administered over 12 months, the vaccinated children in our cohort received just 1 dose because of the early termination of the vaccine program. We have previously reported on overall vaccine immunogenicity and efficacy by BL dengue serostatus for this cohort (24). Here, we focus on how the vaccine performed in children who were DENV seronegative at BL (11% of the cohort) with an emphasis on individual-level vaccine responses and their relationship to vaccine efficacy and safety.

## Results

Paired blood samples collected at BL and the end of study period 1 (P1) were available from 222 BL seronegative children (136 vaccinated and 86 not vaccinated) for the study (Figure 1). Among children in the vaccine and no vaccine groups, the sex distribution and residence were similar, and the mean age at enrollment was approximately 1 year higher in vaccinees (Supplemental Table 1; supplemental material available online with this article; https://doi.org/10.1172/jci.insight.200741DS1).

### Vaccine responses and WT DENV infections during P1 (months 0–20).

Dengvaxia is a tetravalent chimeric YFV/DENV vaccine consisting of the premembrane (PrM) and envelope (E) protein coding regions of each DENV serotype, replacing prM and E segments in the YFV-17D live virus vaccine (22). All the nonstructural viral proteins produced by this vaccine are derived from YFV and not DENVs. We relied on specific antibodies to YFV nonstructural protein 1 (NS1) and DENV1-4 NS1 proteins to detect immune responses stimulated by vaccination only, WT DENV infections only, or both. We first tested paired blood samples collected at BL and P1 for binding antibodies to NS1 from YFV and DENV1-4 and neutralizing antibodies to the 4 DENV serotypes.

During the first study period, 50% (43 of 86) of the children in the no-vaccine group experienced WT DENV infections (Table 1). Since primary DENV infections are characterized by higher levels of binding and NAbs to the infecting serotype compared with heterologous serotype, we were able to identify the serotype responsible for most infections in the unvaccinated group. All 4 serotypes cocirculated in our study population, with DENV2 being the most common (40%), followed by DENV1 (23%), DENV4 (9%), and DENV3 (5%) (Supplemental Table 2). For 10 (23%) children experiencing a new DENV infection, we were unable to determine the serotype. We conclude there was a high force of DENV transmission in Cebu between BL and P1 with cocirculation of all serotypes.

Among vaccinated children, 83 of 136 tested positive for YFV NS1 Ab, demonstrating a 61% vaccine response rate after a single dose (Table 1). Nine vaccinated children developed DENV NAb without seroconverting to YFV or DENV NS1 antigens. These 9 children were classified as vaccine responders, resulting in a total of 92 of 136 (68%) vaccine responders (Table 1). A total of 36 vaccinated children seroconverted to DENV NS1, indicating that 26% of vaccinated children experienced a breakthrough WT DENV infection during P1. These breakthrough infections were similarly distributed (27% verus 25%) between children with or without a detectable vaccine response (Table 1). As 26% (36 of 136) and 50% (43 of 86) of children in the vaccine and no vaccine groups experienced WT DENV infections, vaccine efficacy against WT DENV infection by any serotype was 52% during P1.

### DENV neutralizing antibodies in vaccine and no-vaccine groups.

**Figure 2:**
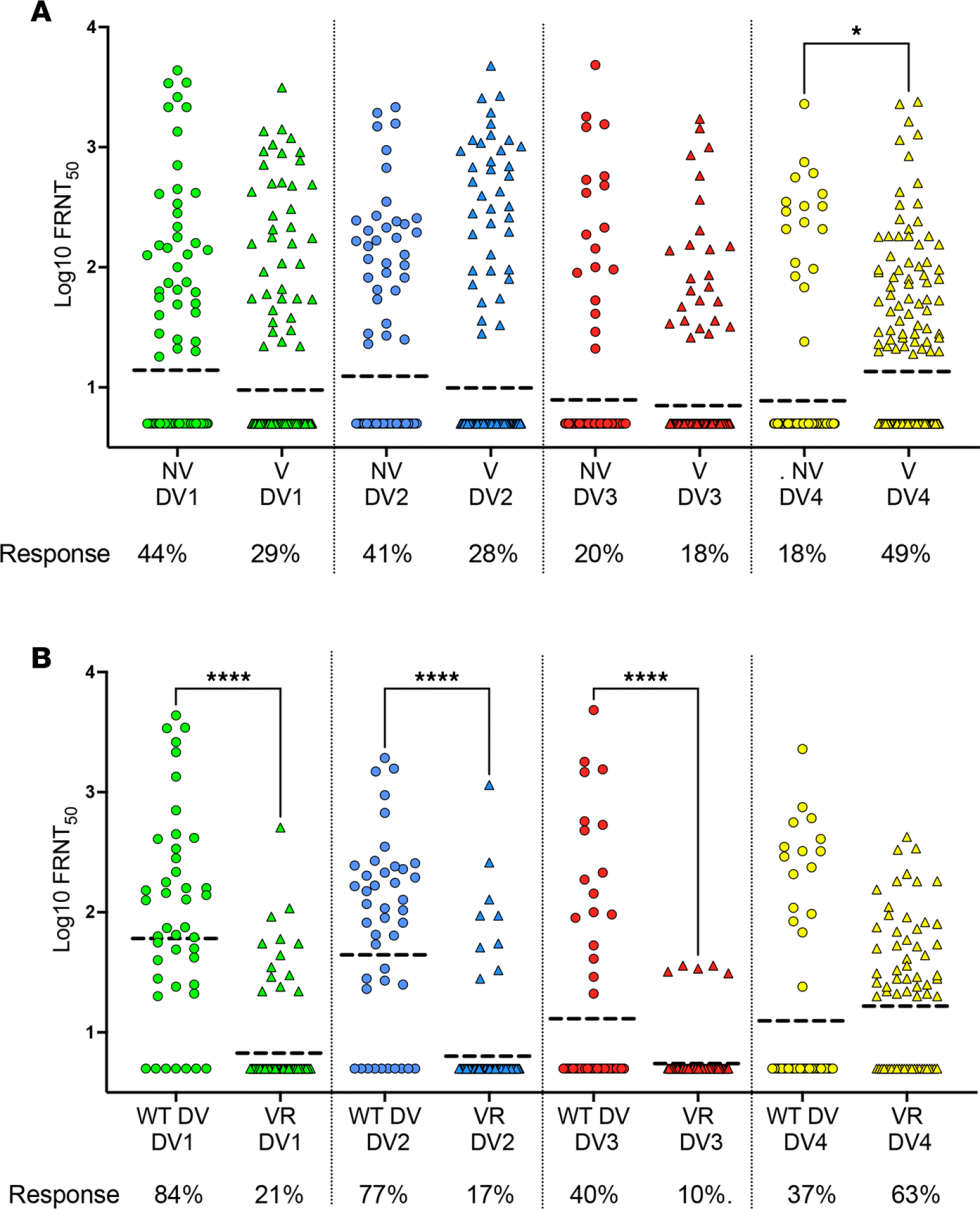
DENV1-4 NAb levels in dengue seronegative children who did or did not receive a single dose of Dengvaxia. (**A**) DENV1-4 NAb responses at the end of P1 in all children with no vaccine (NV) (*n* = 86) or vaccine (V) (*n* = 136). (**B**) DENV1-4 NAb responses in unvaccinated children exposed to a WT DENV infection (WT DV) (*n* = 43) and vaccinated children with a positive vaccine response only (VR) (N = 67). Statistical analysis done by 1-way ANOVA. *P = 0.0417, ****P < 0.0001. GMT indicated as a dotted line. The percentage of children that responded (FRNT_50_ ≥ 20) to each serotype are indicated.

To characterize DENV NAbs stimulated by the vaccine, we measured levels of NAbs to each serotype at the end of P1 (Figure 2). For the neutralization assay, we used fully mature, low-passage, clinical strains of each serotype because partially mature, laboratory-adapted strains of DENVs routinely used in this assay are sensitive to neutralization by some antibodies that are unlikely to be protective in humans (25–27). The mean titers of NAb to DENV1, -2, and -3 were similar between vaccinated and unvaccinated children (Figure 2A). The DENV4 NAb response was higher (in frequency and magnitude) in vaccinated compared with unvaccinated children (Figure 2A).

Among children in the vaccine arm, NAbs measured at P1 may be derived from the vaccine, WT DENV infections, or both. To characterize NAbs stimulated by vaccination only, we restricted our analysis to 67 vaccine responders (YFV NS1^+^) who did not have breakthrough WT DENV infections during P1. Nearly 30% (20 of 67) of children with a vaccine response had no detectable NAb at P1 (Supplemental Table 3). Among vaccine responders with DENV NAb, 31 of 47 (66%) had a monotypic NAb response to DENV4 (Supplemental Table 3).

Next, we compared DENV NAb responses in children who responded to the vaccine only (*n* = 67) and unvaccinated children with WT DENV infections (*n* = 43) during P1 (Figure 2B). Vaccine responders had lower NAb responses to DENV1, -2, and -3 compared with unvaccinated children exposed to WT DENV infections. The vaccine stimulated DENV4 responses more frequently than WT DENV infections (63% verus 37%) (Figure 2B). Collectively, these results demonstrate the replication and immunodominance of the DENV4 vaccine component over the other 3 serotype vaccine components in Dengvaxia.

### Vaccine efficacy against virologically confirmed dengue disease (VCD).

As previously reported (24), children in the Cebu Dengvaxia cohort were prospectively monitored for 5 years to capture instances of symptomatic dengue. Over the entire 5-year follow-up period, the vaccine did not protect BL seronegative children from VCD (Supplemental Table 4) (24). In fact, a higher proportion of cases occurred among vaccinated (19.1%) compared with unvaccinated (12.8%) children, although this difference was not statistically significant (Supplemental Table 4).

**Figure 3:**
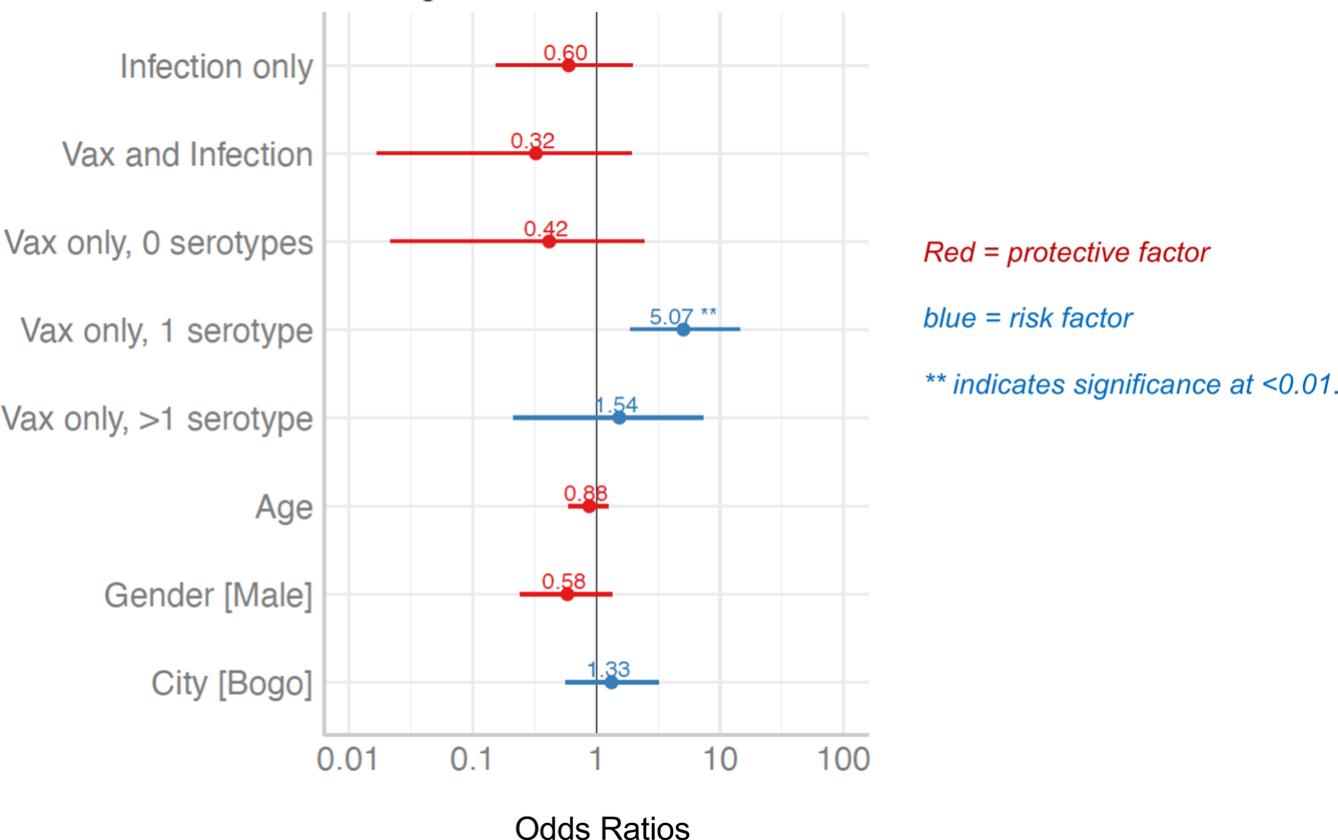
Logistic regression model. A logistic regression model (adjusted for age, sex, and location) was performed to evaluate the probability of having a dengue breakthrough case during P1–P5 for the following groups: remained naive (reference group), had a WT infection, had a vaccine response and a WT infection, or had a vaccine response that neutralized 0, 1, or 2 or more serotypes. The odds ratios and corresponding 95% CI are shown. The only group that had a significant increased risk for being a VCD patient compared with the reference group was the vaccine only responders with a NAb response to 1 serotype only. Supplemental Table 5 displays the number of VCD cases in each group.

Most dengue seronegative children enrolled for the Cebu Dengvaxia study acquired some level of immunity to DENV during P1 as a consequence of vaccination and/or WT DENV infection. We used a logistic regression model to evaluate if vaccine, WT DENV infection, and serotype-specific NAb status at the end of P1 altered the probability of having a dengue breakthrough case between P1 and the end of the study (P5) (Figure 3 and Supplemental Table 5). For this analysis, we used unvaccinated seronegative children who remained DENV naive at P1 as the reference group. We observed that vaccinated children with a NAb response to 1 serotype only were at greater risk of being a case compared with the DENV-naive control group (OR, 5.07, *P* = 0.002). This elevated risk was not observed in children who responded to the vaccine (YFV NS1 Ab positive) but failed to develop or maintain NAb response through P1 (Vax only, 0 serotypes) or children who had vaccine-induced NAb responses to more than 1 serotype (Vax only, > 1 serotype) at P1.

## Discussion

From Dengvaxia clinical trials, the scientific community learned that an imbalanced tetravalent live vaccine dominated by the replication of 1 vaccine serotype was not safe to use in dengue seronegative children (19). We sought to characterize Ab responses in seronegative children who received the vaccine and to identify responses that correlated with vaccine-enhanced WT DENV infections.

During the 20-month span of our study, 50% of unvaccinated children experienced WT DENV infections. Given this high attack rate, we relied on assays that measured antibodies to YFV and DENV NS1 proteins to distinguish Ab responses stimulated by the vaccine and WT DENV infections. Since flavivirus NS1 proteins have regions that are conserved across the family and unique to each virus species, assay cutoff values were set to minimize false-positive results, especially WT DENV infections being misclassified as vaccine responses. Using the cohort of unvaccinated children with a 50% attack rate of WT DENV infections, we determined that the YFV NS1 Ab assay had a false positive rate of 8% (4 of 50 samples tested). Thus, it is likely that a few children in the vaccine arm designated as vaccine responders + WT DENV infections reflect false-positive vaccine responses.

NAbs have been used as a correlate for guiding DENV vaccines. However, in clinical trials with leading vaccines, the presence or level of NAb to a serotype (especially in seronegative children) was not a reliable predictor of protection (8, 9, 22). Recent studies have demonstrated that laboratory-adapted, partially mature DENV virus strains commonly used in the NAb assay are sensitive to neutralization by antibodies that are not protective in vivo (25–28). We used fully mature clinical strains for each serotype in the NAb assay because mature virions are less susceptible to neutralization by antibodies that are not protective in vivo (25–27). The NAb response in vaccinated children was strongly biased in favor of DENV4, confirming the immunodominance of the DENV4 vaccine component in Dengvaxia and the frequent failure of other vaccine components.

We used paired serology to assess the effect of vaccination on all DENV infections (asymptomatic and symptomatic infections) during P1. Salje et al., used a similar approach with 611 children in the Philippines enrolled in Sanofi’s phase III Asian clinical trial to estimate vaccine efficacy against symptomatic and subclinicial infections (29). They reported vaccine efficacy against asymptomatic and symptomatic infections over a period of 3–5 years in BL seropositive children who were vaccinated. There were too few seronegative children in their study to determine vaccine efficacy against infection in this population. We enrolled a sufficient number of seronegative children to assess vaccine-mediated protection. During P1, the vaccine reduced the number of WT DENV infections by nearly half (vaccine efficacy against infection = 52%). Primary WT DENV infections are known to produce a period of transient serotype cross-protection for 1–2 years (30). We suspect that a similar immune mechanism is responsible for transient cross-protection in response to this DENV4-dominant and imbalanced live virus vaccine.

We were surprised by the absence of vaccine-specific YFV NS1 antibody and DENV NAb in 32% of vaccinated children at the end of P1. Moreover, 22% of children designated as vaccine responders had a response to YFV NS1 but no detectable DENV NAb at month 20. Some “non/ poor-responders” may represent true vaccine failure, while others may have developed responses that waned to undetectable levels by 20 months post vaccination. Our observation that both vaccine responders and “nonresponders” experienced a lower frequency of WT DENV infections in P1 compared with unvaccinated children suggests that failure to observe a response is most likely due to waning immunity. While the children in our study received only a single vaccine dose, Dengvaxia was approved as a 3-dose vaccine administered over 1 year, in part, to increase responses in children who failed to respond after the first or second dose (22).

In Dengvaxia clinical trials, seronegative vaccinated children had a 2- to 4-fold elevated risk of experiencing a clinically severe dengue infection compared with unvaccinated children (12). As the original trial was not designed to separately monitor BL seronegative and positive participants, investigators were unable to analyze specific vaccine responses in seronegative children and their relationship to subsequent severe WT DENV infections. We observed that seronegative vaccinated children who developed a monotypic NAb response had a 4.8-fold elevated risk for experiencing a VCD compared with unvaccinated children. As vaccinated children with no NAb or NAb to 2 or more serotypes did not have the same risk, our results pinpoint the presence of durable NAb to just 1 serotype as a strong predictor of vaccine-enhanced dengue disease. These individuals are likely to have an abundance of serotype cross-reactive, nonneutralizing antibodies implicated in the enhanced replication of heterologous serotypes. We propose that primary DENV infections or imbalanced live vaccines enhance subsequent heterologous WT infections by a shared mechanism triggered by cross-reactive, non-NAbs.

Our results have implications for evaluating other leading dengue vaccines or vaccines in development. Many studies have established that Qdenga, the second leading tetravalent live dengue vaccine, is imbalanced and vaccine immunity is mainly, if not exclusively, driven by the serotype 2 vaccine component (7, 9, 18). While the overall number of dengue cases during the last 3 years of the QDenga phase III trial was insufficient to assess safety in seronegative individuals, of particular concern was a trend of more hospitalized DENV3 cases among vaccinated children compared with unvaccinated seronegative children (6). Despite these shortcomings, some regulatory agencies, including the WHO, have recommended using this vaccine in seronegative children, while at the same time acknowledging the need for more safety data (15). Our finding that a vaccine-induced monotypic NAb response is a predictor of subsequent vaccine-enhanced WT dengue disease can be used to flag vaccine candidates that are likely to pose a special risk to seronegative patients and require extensive safety data before use in this population.

## Methods

### Cohort information.

A cohort of 2,996 children (ages 9–14) was established by Ylada et al. in 2017 (24) in Cebu, Philippines, and prospectively followed for 5 years to study the immunogenicity and efficacy of Dengvaxia by BL dengue serostatus and examine dengue risk among those who were eligible to receive the vaccine. While Dengvaxia was approved as a 3-dose vaccine administered over 12 months, the vaccinated children in our cohort only received a single dose because the vaccination program was discontinued when Sanofi reported that the vaccine was contraindicated in seronegative children. At the start of the study, 60% received 1 dose of Dengvaxia, and the remaining 40% were not vaccinated. Blood samples were collected from the entire cohort at BL and yearly at the indicated follow-up times (P1–P5) to measure vaccine immunogenicity and to capture all dengue infections (asymptomatic and symptomatic) by serology (Figure 1). The year-1 samples (P1) were collected at a median of 20 months (IQR, 17–22). The cohort was monitored for acute febrile illness (AFI) to capture symptomatic dengue cases. All participants had basic demographic information collected, and AFI were evaluated with a case report form and reverse transcriptase PCR (RT-PCR) for DENV1-4.

### Sample selection for current study.

BL dengue immunity was evaluated in all individuals by indirect DENV IgG ELISA (PanBio; Brisbane, QLD, Australia) and confirmed by standard reference PRNT (31). As previously described, sera at a single dilution of 1:40 that failed to neutralize 70% or more of the inoculum of any serotype in a focus reduction neutralization test (FRNT) with the 4 DENV serotypes was determined to be DENV seronegative as BL (31). Of the 2,996 children in the cohort, 320 (11%) were dengue seronegative at BL based on serology. Of those, 181 received 1 dose of Dengvaxia, and 139 remained unvaccinated. Paired serum samples collected at BL and FP1 (18 months after vaccination, range 15–22 months) from 222 (vaccine = 136; no vaccine = 86) seronegative children were analyzed for the current study.

### DENV1-4 focus reduction neutralizing test.

DENV serotype-specific neutralizing antibody levels were measured using a FRNT against mature virions as described previously (23, 28). Here we used mature forms of low-passage clinical isolates that matched the 4 dengue genotypes circulating in the Philippines during the study period (GenBank accession nos. AIE17470.1, QBP33521.1, AFI55000, and AHN50410). Fully mature dengue virions were produced in our laboratory by infecting Vero-81 cells overexpressing human furin (32). Briefly, heat-inactivated serum was serially diluted 3-fold over a range from 1:20 to 1:20,480 and mixed with a constant amount (60–90 foci) of each of the 4 dengue virus serotypes and incubated for 1 hour at 37°C. The mixtures were added to duplicate wells of confluent Vero-81 cells (ATCC CCL-81) in a flat-bottom 96-well plate (Corning Costar, 3598) and incubated for 1 hour at 37°C. After adsorption, cells were overlayed with 1% carboxymethylcellulose (Sigma, M0512) and incubated at 37°C for 48 hour, then fixed in 2% PFA diluted in PBS, blocked in 5% nonfat dried milk and immunostained with flavivirus cross-reactive mouse monoclonal antibodies anti-E 4G2 (ATCC HB-112; 0.8 mg/mL, Lofstrand Labs, Gaithersburg, MD, USA) or anti-prM 2H2 (ATCC HB-112; 1.63 mg/mL, Lofstrand Labs, Gaithersburg, MD, USA), or both, for 1 hour at 37°C. To visualize DENV-infected cells, wells were incubated for 1 hour at 37°C with horseradish peroxidase–labeled (HRP-labeled) goat anti–mouse IgG secondary antibody (Jackson ImmunoResearch Laboratories) and developed using TrueBlue HRP substrate. Images of all wells were collected using the Cellular Technology Limited (CTL) machine and ImmunoSpot DC software for automated plaque counting and QC. Foci were counted in each well and compared with foci count in control virus only wells. FRNT_50_ values were calculated by graphing percentage of neutralization verus serum dilution and fitting a sigmoidal dose response (variable slope) using Prism 9 (GraphPad Software, San Diego, CA, USA) and represent the dilution at which the serum neutralizes 50% of the infection. Log-transformed data from FRNT_50_ values were used to calculate GMT ± 95% CI. EC_50_ < 20 was considered negative and tabulated as 10.

### Luminex multiplex assay with DENV1-4 EDIII, DENV1-4 NS1, and YFV NS1.

Antibody responses to E protein domain 3 (EDIII) of DENV serotypes 1-4 and nonstructural 1 protein (NS1) of YFV and DENV serotypes 1-4 were measured using Luminex multiplex assay as previously described (33). Briefly, biotinylated EDIII antigens and biotinylated bovine serum albumin (BSA) were coupled to unique MagPlex-Avidin Microspheres (Luminex) while His-tagged NS1 antigens (The Native Antigen Company) were coupled following immobilization of anti-His tag antibody (abcam) onto unique avidin-coated microspheres. The panel of EDIII, NS1 and BSA conjugated microspheres were mixed in equal ratios and plated at 2,500 beads per antigen in 50 μL/well in 96-well plates. Diluted human serum (1:500) was incubated with antigen-conjugated microspheres for 1 hour at 37°C (700 rpm). Later, immune complexes were incubated with goat anti–human IgG Fc multispecies SP ads-PE antibody (Southern Biotech) following 3 washes. Antibody responses were detected using a Luminex 200 analyzer and expressed as median fluorescence intensity after subtracting the nonspecific antibody binding signal (to BSA). Selected samples from healthy donors and well-characterized DENV and ZIKV seropositive individuals were run on multiple assay plates to verify assay performance and assess interassay variability.

### Anti-YFV NS1 IgG ELISA assay.

Flat-bottom 96-well microtiter plates (BioOne Microlon, Greiner) were coated with recombinant YFV NS1 antigen, obtained commercially from Native Antigen Company, Oxfordshire, UK, at a concentration of 1 μg/mL in carbonate/bicarbonate buffer pH 9.6. After overnight incubation at 4°C, plates were washed with PBS + 0.05% Tween-20 (PBS-T) and blocked with PBS-T supplemented with 3% (v/v) goat normal serum (X% GNS; Gibco, Gaithersburg, USA) for 60 minutes at 37°C. The plates were washed again with PBS-T; then, serum samples diluted 1:20 in blocking buffer (PBS-T 3% BSA) were added to duplicate wells and incubated for 60 minutes at 37°C. Plates were washed with PBS-T and incubated for 1 hour at 37°C peroxidase-conjugated F (ab’)2 goat anti-human IgG, Fcγ Fragment (Jackson ImmunoResearch, West Grove, USA), diluted 1: 2500 for AP in blocking buffer. The plates were washed again with PBS-T and color was developed with SureBlue Reserve TMB Microwell Peroxidase Substrate (SeraCare, Milford, USA). Optical density at 450 nm (OD_450_) was monitored for 20 minutes and recorded for all wells once the OD_450_ of positive control DT281a (flavi naive, YFV Vax) reached 1.0, using the Microplate reader (Molecular Devices, Sunnyvale, USA).

Human positive (DT281a) and negative control (NHS) sera were run in every plate. Yellow fever–antibody positive human serum samples obtained from healthy adult donors who received YFV vaccine and were DENV and ZIKV naive were provided by M. Collins at Emory University, Atlanta, Georgia, USA. A high assay cutoff value ≥ 0.7 (OD_450_ at a serum dilution of 1:20) was selected to minimize false-positive signals from recent DENV infections.

### Vaccine responses (BL to P1).

As Dengvaxia is a chimeric DENV vaccine on a YFV backbone encoding for YFV nonstructural proteins, we relied on specific antibodies to YFV NS1 protein as a marker of vaccine replication and immunogenicity. Paired BL and FY1 blood samples from unvaccinated and vaccinated children were tested for YFV NS1 antibodies by ELISA and the multiplex serology assay. Assay cutoff values were selected to minimize false-positive signals from recent DENV infections. A positive response for YFV NS1-specific Ab in the ELISA and/or multiplex serology assay was considered evidence of vaccine replication and immunogenicity. Using these criteria, the false-positive rate among unvaccinated children was 8% (4 of 50 children tested were positive).

### New DENV infections (BL to P1).

Paired blood samples collected at BL and FP1 from each child were tested to capture new DENV infections. In the multiplex serological assay, any patient who seroconverted to NS1 from one or more DENV serotype was considered to have experienced a primary DENV infection. For the no vaccine group, the serotype responsible for infection was determined using the levels of NAbs to each serotype or the magnitude of binding antibodies to EDIII and NS1 antigens from each serotype. Individuals with NAb to one serotype only or a NAb titer to one serotype that was 3 times higher than the titer to any other serotype was considered to have experienced an infection with the highest titer serotype. If the NAb titer was inconclusive, we relied on levels of DENV1-4 EDIII or NS1 binding antibodies in the multiplex Luminex assay to determine serotype (EDIII and/or NS1 Ab response positive for a single serotype or the signal to one serotype being 4-fold higher than any other serotype). If both the neutralization and multiplex serological assays were inconclusive, the serotype of infection was unknown. We did not try to ascertain serotypes responsible for vaccine breakthrough infections because these infections, often, stimulated secondary immune responses, which do not reliably reveal the serotype of infection.

### Statistics.

Analyses were conducted using R version 4.3.3 (https://www.R-project.org/). We used logistic regression to estimate the odds of experiencing dengue for the following groups: remained naive (reference group), had WT infection, had a vaccine response and WT infection, or had a vaccine response and neutralized 0, 1, or 2 or more serotypes. All analyses included age, sex, and location to account for relevant demographic covariates. The log-odds of experiencing dengue verus no dengue was estimated using the glm function in the stats package in R, assuming a binomial distribution and using the logit link function. The odds ratios and corresponding 95% confidence intervals were visualized using the plot_model function in the sjPlot package (https://CRAN.R-project.org/package=sjPlot). Of note, WT infection group included all with evidence of WT infection, both those in the unvaccinated group and those in the vaccinated group but without a vaccine response. Two individuals experienced 2 dengue cases during follow up; for these analyses, only the first case was modeled.

### Study approval.

The study protocol was approved by the University of the Philippines – Manila Research Ethics Board (UPM REB 2016-435-01) and is registered at clinicaltrials.gov (NCT03465254). Parents or guardians provided written informed consent, and children provided documented oral assent before participation in the study, in accordance with local regulations.

### Data availability.

Values for all data points in graphs are reported in the Supporting Data Values file.

## Author contributions

ALL, MY, and JD conceived the cohort study and wrote the study protocol. MY, MVC, JVD, KAA, and JD implemented the field activities. JVD trained the staff on sample collection, storage and transportation. CA carried out the standard plaque reduction neutralization testing on BL samples to establish an immune profile and implemented the YFV-NS1 ELISA for testing vaccine response. LJW, AMD, CA, and ALL designed and planned the study. EA, EF, RS, and LJW carried out the paired immunogenicity testing of BL and follow-up samples using the mature plaque reduction neutralization test. LDH and LP designed and performed the Luminex assays to determine DENV 1-4 NS1 and YFV NS1 antibodies. LK conceived and performed the immune correlates analyses. LJW and AMD wrote the first draft of the manuscript. LJW, AMD, LK, MAF, JD, MY, and JVD, reviewed and edited the manuscript. LJW, AMD, LDH, LK, and MAF contributed to the analysis and interpretation of the data and the final draft of the paper.

## Conflict of interest

The authors have declared that no conflict of interest exists.

## Funding support

This work is the result of NIH funding, in whole or in part, and is subject to the NIH Public Access Policy. Through acceptance of this federal funding, the NIH has been given a right to make the work publicly available in PubMed Central.

- US National Institutes of Health grants 2P01AI106695 and U19AI181960.
- The Intramural Research Program of the US National Institutes of Health.
- The Philippine Department of Health.
- Hanako Foundation.
- Swedish International Development Cooperation Agency.
- International Vaccine Institute.

## Supplementary Material

Supplemental data

Supporting data values

## Figures and Tables

**Table 1:**
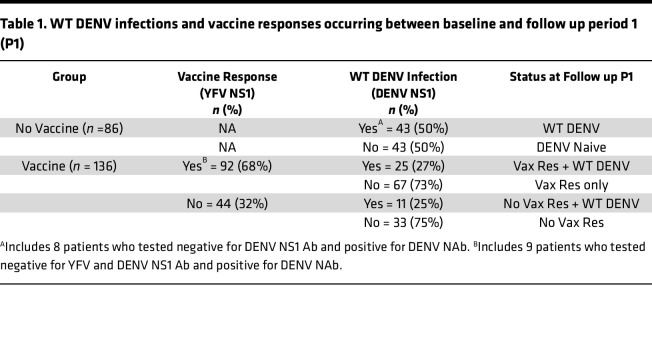
WT DENV infections and vaccine responses occurring between baseline and follow up period 1 (P1)

## Version 1 03/17/2026

In-Press Preview

## Version 2 04/22/2026

Electronic publication
