# Supplementary material for: Safety of a tetravalent live dengue virus vaccine in children responding to one serotype only
Source: JCI Insight. 2026 Mar 17;11(8):e200741. doi: 10.1172/jci.insight.200741 (PMC13135402; doi:10.1172/jci.insight.200741)
Supplement: Supplemental data [file jciinsight-11-200741-s061.pdf]

Supplementary Table 1. Comparison of baseline seronegative participants selected for study who received one dose and no dose of CYD-TDV (N=222)

Characteristics	Vaccinated (N=136)	Not vaccinated (N=86)	Total (N=222)	p-value Fischer's exact test
Age (years) at enrollment				
Mean (SD)	11 (1.28)	10 (1.12)	10 (1.23)	0.0335
Sex				
Female N (%)	72 (53)	46 (54)	118 (53)	>0.9999
Male N (%)	64 (47)	40 (46)	104 (47)	
Residence				
Bogo city N (%)	73 (54)	41 (48)	114 (51)	0.4103
Balamban N (%)	63 (46)	45 (52)	108 (49)	

Supplementary Table 2. DENV serotypes* responsible for Infection during study period 1 (BL to P1) in unvaccinated children (N = 43)

DV1 (%)	DV2 (%)	DV3 (%)	DV4 (%)	Unknown (%)
10 (23)	17 (40)	2 (5)	4 (9)	10 (23)

*RULES FOR SEROTYPING

1) At P1 NAb to one serotype only or 3X higher NAb to one ST compared to others

2) If NAb is inconclusive, Luminex EDIII and/or NS1 response to one ST only

3) If NAb and Luminex are both inconclusive, then serotype listed as “Unknown”

Supplementary Table 3. Number of children that neutralized 1 serotype, 2 or more serotypes, or none after WT DENV infections and/or vaccination during Follow Up Period 1

Group	Exposure (N)	DENV1-4 NAb		
		No NAb	NAb to 1 Serotype	NAb to 2+ Serotype
No Vaccine (N =86)	WT DENV Infection (43)	1	9	33
	No WT DENV Infection (43)	43	0	0
Vaccine (N = 136)	Vaccine Response + WT DENV Infection (25)	3	1	21
	Vaccine Response only (67)	20	34[A]	13
	No Vaccine Response + WT DENV Infection (11)	1	3	7
	No Vaccine Response + No WT DENV Infection (33)	33	0	0

[A]Among the 34 vaccine responders only with monotypic NAb responses, 31 neutralized DENV4.

Supplementary Table 4. Virologically Confirmed Dengue Cases (VCD) in Vaccinated vs Not Vaccinated children . monitored for VCD cases occurring over the entire course of the study from BL to P5.

Vaccine Status	N	Cases (%)	DENV Serotypes	Relative Risk of being a case after vaccination (95% CI) Fishers Exact Test
Not Vaccinated	86	11 (12.8%)	DV1 = 4; DV2 = 3; DV3 = 2; DV4 = 1; Unknown = 1	RR = 1.49 (0.8 - 2.9)
Vaccinated	136	26 (19.1%)	DV1 = 4; DV2 = 10; DV3 = 8; DV4 = 0; Unknown = 4.	

Supplementary Table 5. VCD Cases (P1-P5) by vaccine response (stratified by NAb) and/or WT DENV infection status used for regression model and risk analysis (Figure 3)

Group	Vaccine and WT DENV Infection Status at Study Period 1 (P1)	DENV NAb at P1	N	DENV Cases (%)
No Vaccine (N = 86)	*Naïve (No WT DENV infection at P1)*	None	43	7 (16.2%)
	Primary DENV Immune (WT DENV infection at P1)	Monotypic:22%<br>Multitypic: 76%	43	2 (4.7%)
Vaccine (N = 136)	Vaccine Response (YFV NS1 Ab +) only *(No WT DENV infection at P1)*	None	20	1 (5.0%)
		Monotypic	34	13 (38.2%)
		Multitypic	13	2 (15.4%)
	Vaccine Response (YFV NS1 Ab +) and Breakthrough WT DENV infection at P1	Monotypic: 14%<br>Multitypic: 82%	25	1 (4.0%)
	Vaccine Failure (YFV NS1 Ab -) and no WT DENV infection at P1	None	33	2 (6.1%)
	Vaccine Failure (YFV NS1 Ab -) and WT DENV infection at P1	Monotypic: 30%<br>Multitypic: 70%	11	2 (18.2%)
